# Coding of spatial attention priorities and object features in the macaque lateral intraparietal cortex

**DOI:** 10.14814/phy2.13136

**Published:** 2017-03-07

**Authors:** Ekaterina Levichkina, Yuri B. Saalmann, Trichur R. Vidyasagar

**Affiliations:** ^1^ Department of Optometry & Vision SciencesUniversity of MelbourneMelbourneAustralia; ^2^ Institute for Information Transmission ProblemsRASMoscowRussia; ^3^ Department of PsychologyUniversity of Wisconsin ‐ MadisonMadisonWisconsin; ^4^ Melbourne Neuroscience InstituteUniversity of MelbourneAustralia; ^5^ Department of Anatomy & NeuroscienceUniversity of MelbourneMelbourneAustralia

**Keywords:** Area LIP, delayed match‐to‐sample task, orientation selectivity, Parietal cortex, visual attention

## Abstract

Primate posterior parietal cortex (PPC) is known to be involved in controlling spatial attention. Neurons in one part of the PPC, the lateral intraparietal area (LIP), show enhanced responses to objects at attended locations. Although many are selective for object features, such as the orientation of a visual stimulus, it is not clear how LIP circuits integrate feature‐selective information when providing attentional feedback about behaviorally relevant locations to the visual cortex. We studied the relationship between object feature and spatial attention properties of LIP cells in two macaques by measuring the cells' orientation selectivity and the degree of attentional enhancement while performing a delayed match‐to‐sample task. Monkeys had to match both the location and orientation of two visual gratings presented separately in time. We found a wide range in orientation selectivity and degree of attentional enhancement among LIP neurons. However, cells with significant attentional enhancement had much less orientation selectivity in their response than cells which showed no significant modulation by attention. Additionally, orientation‐selective cells showed working memory activity for their preferred orientation, whereas cells showing attentional enhancement also synchronized with local neuronal activity. These results are consistent with models of selective attention incorporating two stages, where an initial feature‐selective process guides a second stage of focal spatial attention. We suggest that LIP contributes to both stages, where the first stage involves orientation‐selective LIP cells that support working memory of the relevant feature, and the second stage involves attention‐enhanced LIP cells that synchronize to provide feedback on spatial priorities.

## Introduction

The lateral intraparietal area (LIP), a part of the macaque posterior parietal cortex, has long been recognized as a brain region essential for the control of attention (Colby and Goldberg 1999; Bisley and Goldberg 2003). It possesses a saliency or priority map (Walther and Koch 2006; Bisley and Goldberg 2010), which is believed to represent spatial locations in the visual field that are preferentially highlighted either by bottom‐up sensory inputs or top‐down signals from the prefrontal cortex. The suggestion that the map is fundamental for selection of the focus of attention (Bisley et al. 2011) is supported by evidence of LIP neurons providing spatial attention signals that modulate responses in earlier visual areas such as area V5/MT (Saalmann et al. 2007). Such feedback from dorsal cortical areas has been postulated to play a pivotal role (Vidyasagar 1999; Bullier 2001; Deco and Rolls 2004) in gating sensory inputs as early as area V1, where attention‐related responses have been identified in both macaque (Vidyasagar 1998; McAdams and Reid 2005) and human (Brefzynski and DeYoe 1999; Gandhi et al. 1999) studies.

Though the output from LIP to early visual areas seems to be largely spatial (Saalmann et al. 2007), and spatial cues tend to be dominant over feature‐based cues in search tasks (Verghese et al. 2013), featural information is also necessary for LIP to fulfill its role in selective attention. In many situations, objects sharing a specific feature are selected – for example, all red cars in a car park if the car one is looking for is red – for subsequent serial processing (Treisman and Gormican 1988; Wolfe 1994, 2007). Consistent with this is the evidence for feature selectivity in LIP neurons (Sereno and Maunsell 1998; Sereno et al. 2002; Toth and Assad 2002; Janssen et al. 2008; Ogawa and Komatsu 2009; Swaminathan et al. 2013; Subramanian and Colby 2014). It is possible to deploy spatial attention in one step based on feature‐based signals, if the output from feature‐selective neurons were nonspecifically pooled in direct feedback to early visual cortex. Alternatively, the output of feature‐selective neurons may be integrated within LIP itself in a two‐step process: feature‐selective cells that hold the memory of the sensory input long enough to enable the formation of a saliency map, and output spatial attention cells that dynamically represent salient information to provide spatial feedback to other areas. Thus, one might encounter (at least) two types of LIP cells: one showing feature selectivity and working memory, and the other showing attentional modulation and little feature selectivity, but showing local synchrony that strengthens the output mediating LIP's known spatial attention effects on earlier visual areas (Saalmann et al. 2007). Distinct feature‐selective and spatial cell types have been reported in LIP (Ogawa and Komatsu 2009), although the saccadic responses may have contributed to the observed neural modulation. It is not clear if these cells, respectively, show featural memory and spatial attention‐enhanced local synchrony, important for attentional feedback.

We tested this proposed functional differentiation of cell types by analyzing the orientation selectivity, working memory and degree of attentional modulation of LIP cells while monkeys performed a delayed match‐to‐sample task that involved both featural and spatial components: the first stimulus (sample) draws the monkey's attention to the possible location and feature of the second stimulus (test). Our results indicate that LIP cells that show more attentional enhancement and neural synchrony tend to be poorly tuned to orientation, and those cells that show sharper orientation selectivity and featural memory do not exhibit a strong modulation by attention.

## Materials and Methods

### Animal care and behavioral training

**Figure 1:**
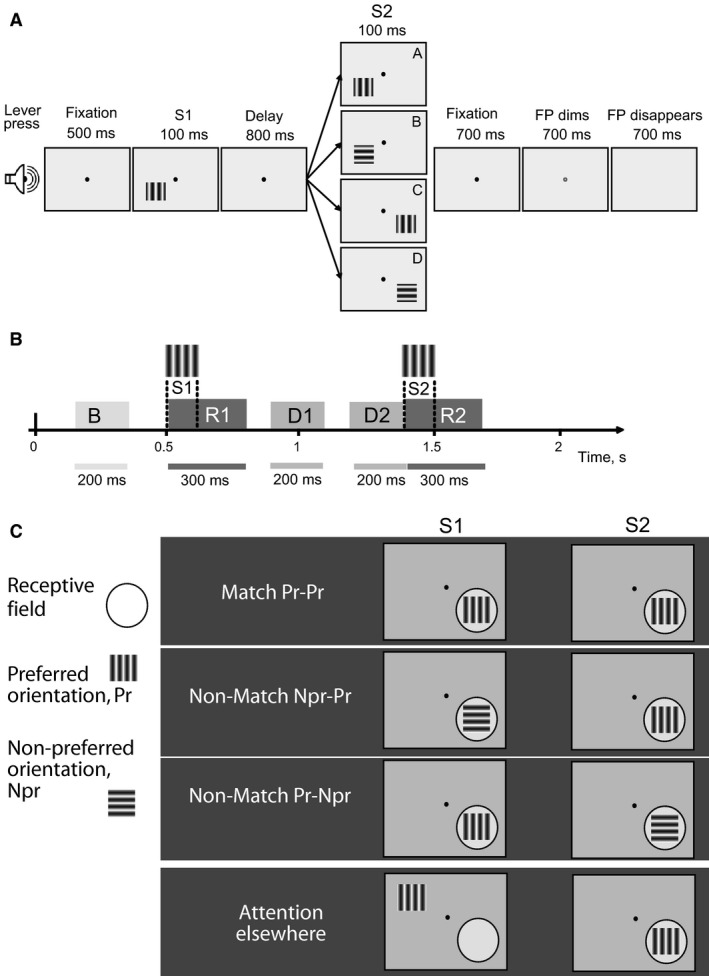
(A) Delayed Match‐to‐Sample Task (DMS)**.** Monkey initiates the trial by pressing the lever and the fixation point (FP) appears at the center of the screen. Monkey needs to maintain fixation during the trial. First stimulus (S1) is presented after 500 msec for 100 msec, and the second stimulus (S2) is presented for 100 msec, 1400 msec after the trial onset, that is, with an 800 msec delay after S1 offset. S1 and S2 could have one of 2 orientations (preferred and orthogonal to the preferred orientations for the cell(s) at the recording site and could appear in 2–5 different locations with one of the locations within the receptive field of the cell and other locations outside the receptive field. Monkey has to match two stimuli by both orientation and location. If the two stimuli match, the monkey releases the lever when FP dims. If they do not match in orientation, location or both, the monkey releases the lever when FP disappears. This arrangement allows testing both spatial and featural attention. **(**B) Time course of the trial and the intervals of interest. B ‐ background activity, R1 ‐ response to the first stimulus, D1 ‐ activity at the beginning of the delay period, D2 ‐ activity at the end of the delay period, R2 ‐ response to the second stimulus. (C) Four types of trials, categorized for analysis. The circle represents the receptive field location of the LIP cell, but the circle itself is not presented. Pr and Npr represent the preferred grating orientation and its orthogonal, respectively. See text for further details.

Two male monkeys (*Macaca nemestrina)* were used for this study, which was approved by the University of Melbourne Animal Experimentation Ethics Committee. They were housed together with ad lib access to water and were trained to come voluntarily into the primate chair for training and recording sessions. These two macaques were the same animals that were used in the study by Saalmann et al. (2007) and the data presented here includes the LIP cells investigated in that study of LIP‐MT interactions together with a number of additional cells. The monkeys had been trained in a visual delayed match‐to‐sample task (DMS, Fig. 1A). The task required matching two successively presented gratings (8^0^×8^0^, 30% contrast with a mean luminance of 15 cd/m^2^) for both the location and the orientation of the gratings on each trial. Up to 5 separate screen positions and 2 orthogonal orientations were alternated in pseudorandom sequence between trials, ensuring that approximately 50% of the trials were ‘match’ trials, where the second grating appeared at the same location as the first and its orientation was also the same as the first grating's. Each trial was initiated by the monkey pressing a lever that led to the appearance of a black fixation spot (FP, 0.1° diameter) at the center of the uniform gray screen, which had a luminance of 15 cd/m^2^. The monkey had to fixate the spot throughout the trial, and with any eye movements of more than 1° (monitored using an infrared oculometer; Dr. Bouis), the trial was aborted. After 500 msec from the start of fixation, the first grating stimulus (S1) was presented for 100 msec. After a delay of 800 msec, the second grating (S2) was presented for 100 msec. The monkey had to maintain fixation for a further period of around 700 msec after S2, after which the FP was dimmed for 700 msec, and then extinguished. In the case of a Match trial, that is, when the location and orientation of the two gratings were the same, the monkey had to release the lever during the dimming period, while for any Non‐Match trial, the monkey had to release it after the disappearance of the FP. Each correct response was rewarded with fruit juice.

We specifically employed a task that did not involve saccades as the response and also separated the motor response time by a long interval after the stimulus (at least 700 msec). This design is different from most other experiments on LIP, including that by Ogawa and Komatsu (2009), who also investigated the different functional cell types in LIP. We thus avoided any confounding of the response by saccades which are known to influence LIP responses (Andersen and Buneo 2002). By adopting a delayed match‐to‐sample paradigm, we could also distinguish feature‐based and spatial attention aspects of LIP responses from their feature sensitivity by analyzing the responses to the sample (S1) stimulus, about whose location and orientation the monkey had no knowledge before its presentation. Another advantage of the paradigm was the insight we could gain about neuronal processing during the delay period.

After completion of the training, the macaques were implanted with an aluminum frame as per the “halo technique” (Saalmann et al. 2007; Pigarev et al. 1997, 2009). For further details of the training and surgical procedures, please refer to Supplementary Online Material of Saalmann et al. (2007).

### Electrophysiology

Electrode penetrations into the LIP were guided by structural MRI images of the brain acquired prior to recording sessions. We performed stereotactic craniotomies (2.5 mm diameter), through which we lowered electrodes into LIP. We confirmed LIP recording sites based on the known response characteristics of LIP cells, such as peri‐saccadic responses (starting before saccades) and delay period activity in the memory task. Neuronal activity was recorded, using tungsten or platinum–iridium microelectrodes, filtered at 1–4000 or 10–4000 Hz and sampled at 10,000 Hz by the Cambridge Electronic Design Micro1402 data collection system. The recorded signals were band‐pass filtered in the 300–4000 Hz range for spikes and low‐pass filtered up to 250 Hz for the local field potential (LFP). Spikes were sorted using Spike2 (CED) software on the basis of spike amplitude and shape.

### Data analysis

In our earlier study (Saalmann et al. 2007), the sample size of the LIP population was limited by the necessity that the analysis needed to be done on pairs of simultaneously recorded cells from areas LIP and MT that had overlapping receptive fields (RFs) and similar preferred stimulus orientations. Here, we describe properties of a larger population of LIP cells, not being limited by the above requirement. First, we performed spike rate analyses on the dataset of 56 cells from LIP that showed an excitatory or inhibitory response during any part of the task. We compared background activity of each cell to its response to S1 and, among these, 47 cells that showed a significant excitatory response to the visual stimulus on the RF (response to S1 compared with spontaneous activity, Wilcoxon Signed Rank Test, *P *< 0.05) were used for further analysis. We analyzed orientation selectivity and attentional enhancement of these cells to describe the distribution of these properties as well as the relationships between these characteristics. Orientation selectivity and RF location were first tested manually and the stimulus location on the monitor positioned accordingly. The orientations used for the main experiment were the preferred orientation so determined (Pr) and its orthogonal orientation (nonpreferred, Npr). Cells showing little selectivity to orientation were then tested with vertical and horizontal orientations in the main experimental paradigm. For our data analysis, we used only cells recorded in trials with 800 msec delay between the two gratings to be able to analyze the ramping effect in the delay period.

Spike rate was obtained in 10 msec time‐windows without any smoothing. Statistical comparisons were done, using the Wilcoxon Signed Rank test for all paired comparisons and the Wilcoxon Rank Sum test for all nonpaired comparisons. The Bonferroni–Holm correction was applied for multiple comparisons.

From each trial, we gathered response rates in the following epochs (Fig. 1B):

1. Background activity (B in Fig. 1B) – 150 to 350 msec before the onset of the first grating (S1).
2. Response to the first stimulus (R1) – 0 to 300 msec from the onset of S1.
3. Activity at the beginning of the delay period (D1) – 300 to 500 msec after S1 offset.
4. Activity at the end of the delay period (D2) – 600 to 800 msec after S1 offset.
5. Activity during the whole delay period (D) – from the beginning of D1 till the end of D2, so 300–800 msec after S1 offset.
6. Response to the second stimulus (R2) – 0 to 300 msec from the onset of the second grating (S2).

The monkeys had to maintain fixation after S2 offset for at least another 700 msec before they could possibly make the manual response of releasing the lever in the case of Match trials and up to an additional 700 msec in the case of Non‐Match trials. Thus, the manual response is unlikely to have influenced the time period of the cells' responses that we analyzed.

We normalized all responses of each cell with respect to the response to an optimally oriented S1 grating flashed on the receptive field of the cell. The latter was considered a “neutral stimulus”, that is, without a specific spatial or featural attentional component, since S1 could appear at any one of a number of locations and could have any of the two possible orientations. We then grouped the trials into four types, as depicted in Figure 1C:

1. ‘Match Pr‐Pr’: Trials with both S1 and S2 presented on the RF and having the preferred orientation – “spatial and feature‐based attention” condition. This type of trial provides information regarding attentional enhancement. Since both location and orientation of the two gratings are the same, increased cell response for the second stimulus indicates that attention to the first one influences processing of the second (considering that controls such as stimulus repetition under passive viewing conditions showed no change in response).
2. ‘Non‐Match Npr‐Pr’: Trials with both S1 and S2 on the RF, but with S1 being the nonpreferred and S2 the preferred orientation – the “spatial attention” condition. An increased response to S2 (relative to the response to sample S1 of preferred orientation) indicates spatial attention, acting on S2. Please note that in these trials, when seen in isolation, any increased responsiveness to S2 stimuli can reflect both any feature selectivity and any spatial attentional enhancement that the cell may have, a potential confound that will be addressed later.
3. ‘Non‐Match Pr‐Npr’: Trials with both S1 and S2 on the RF, but with S1 being the preferred and S2 the nonpreferred orientation. Note that this condition pits any feature selectivity of the cell against any response enhancement due to focal spatial attention. Thus, if a cell codes more for feature selectivity than for attentional enhancement, the response to the nonpreferred S2 will be less than for the preferred S1. If the attentional effect is more dominant, the response to S2 will be greater than for S1.
4. ‘Attention elsewhere’: Trials with S1 out of the RF and S2 within the RF and having the preferred orientation.

Note that in three of the four conditions above, the S2 stimuli were identical (1, 2 and 4), they being gratings of preferred orientation flashed on the RF. These conditions differed only in their S1 stimuli and thus directly help to distinguish the different conditions under which attentional modulation can occur.

Only recording sessions with more than 80% accuracy for Match and Non‐Match types of trials were included in the analysis. The AE+ and AE– recordings were not different in relation to the behavioral results. There was also no significant difference in the percentage of correct responses between different positions where the gratings were presented. This part of the results was reported previously for the majority of the present sample (Saalmann et al. 2007). The following five measures were then calculated from all the trials for each LIP cell:

1. *Orientation selectivity*: Before recording from an isolated single unit, the preferred orientation of the cell was first determined manually using hand‐held stimuli and the preferred orientation confirmed with computer‐controlled stimuli. The majority of recording sites in LIP showed some degree of preference for a particular orientation, even though the degree of orientation sensitivity varied a lot between individual cells as described later. The orientation so identified and its orthogonal orientation were used as the two stimulus orientations in the DMS task. All the trials, Match or Non‐Match, where the first stimulus (S1) was presented on the receptive field of the LIP cell, were used to estimate the statistical significance of the orientation preference. Not all cells showed a statistically significant (*P *< 0.05) preference for one of the two orientations. However, choosing the orientation with the higher response as the preferred, we calculated an Orientation Sensitivity Index (OSI) for each cell, using the following formula, which yields an OSI value in a 0–1 range:
  $$\text{OSI}=\left({\text{R1}}_{P}-{\text{R1}}_{\mathrm{np}}\right)/\left({\text{R1}}_{P}+\text{R1}\right)$$
  where R1_p_ and R1_np_ are responses to S1 for preferred and nonpreferred orientations, respectively. In calculating OSI, we used only responses to the first stimuli to avoid any attentional effects that may be present in responses to S2.

*Attentional enhancement*: Attentional enhancement (AE), that is, a response to S2 presented on the RF that is greater than to the neutral stimulus, can potentially occur either if S2 were presented at the same location, irrespective of the orientation of S1 grating (spatial attention) or if it were of the same orientation as S1 presented irrespective of its location (feature‐based attention). To estimate attentional enhancement we calculated an Attentional Enhancement Index,
  AEI=(R2‐R1)/(R2+R1)
  where R1 and R2 are responses to S1 and S2 stimuli respectively. If a cell had significant orientation selectivity, for AEI estimation we used only ‘Match Pr‐Pr’ trials, where both S1 and S2 were of the preferred orientation. If the cell did not have significant orientation selectivity, we first calculated the AEIs separately for ‘Match Pr‐Pr’ trials with S1 and S2 of one orientation (nominally preferred) and for ‘Match Npr‐Npr’ trials with S1 and S2 of the orthogonal orientation. No significant opposing trend in attentional enhancement (i.e., AEI* *< 0) for the nominally nonpreferred orientations was observed for the cells which had significant attentional enhancement for the nominally preferred orientation. Therefore data for both orientations were pooled for the cells that did not have significant orientation selectivity. Significance of the attentional enhancement was tested by comparing R1 and R2 (*P *< 0.05), using Match Pr‐Pr trials. Significant AEI values greater than 0 indicate attentional enhancement. We thus divided cells into 2 groups, namely those that showed significant attentional enhancement (AE+) and those that did not (AE–).

*Delay period activity*: LIP neurons have been shown to exhibit a gradual increase in attention‐related neuronal activity in the delay period (‘ramping’), as previously shown in our version of the DMS task (Saalmann et al. 2007). However, delay period activity, especially immediately following the S1 stimulus and continuing through the delay period at a stable level may also represent working memory, as often found in many cortical areas, especially prefrontal and parietal cortices (Fuster and Alexander 1971; Gnadt and Andersen 1988; for review, see Ikkai and Curtis 2011). To study whether general delay period activity and the gradual ramping up of the maintained discharge during the delay period is present in both AE+ and AE– cells, we compared the mean discharge rate in two 200 msec windows toward the start and the end of the delay period. One (D1) started 300 msec after offset of S1 (thereby excluding the S1‐evoked response) and the other (D2), started 600 msec after S1 offset and ending with the onset of S2. Significance of ramping activity in the delay period was tested by comparing activity during D1 and D2 (Wilcoxon Signed Rank test), and an index of delay period ramping (ramping delay index, RDI) was calculated:

RDI=(D2‐D1)/(D1+D2)

To test for a stable, nonramping increase in delay period discharge, we also compared the discharge rate in the whole period from the start of D1 to the end of D2 to the background period (B) prior to S1 as well as responses during each of the D1 and D2 periods to the background, B (Wilcoxon Signed Rank test). Cells with memory‐type activity may have significantly greater response in the delay period without a significant difference between D1 and D2, whereas an attention‐related response may show a gradual increase in activity when approaching S2 in time, as noted in many other studies, including human imaging studies (Ikkai and Curtis 2011). For this analysis, we used all trials when S1 was the preferred stimulus falling on the cell's RF, irrespective of what and where S2 was.

*Spike‐field coherence and spike‐triggered LFP average**:*** Coherence is a widely used measure of synchronization between two neural signals in the frequency domain. To measure the local synchrony between the activity of a single LIP cell in relation to its immediate neighbors, we calculated the spike‐field coherence (Jarvis and Mitra 2001; Pesaran et al. 2002; Saalmann et al. 2007). Previous work suggests that attention synchronizes the output from LIP neurons to visual cortex (Saalmann et al. 2007). If there is a two‐stage process in LIP leading to the synchronized output, the spike‐field coherence during the delay period prior to S2 should be more apparent among the AE+ cells rather than in the AE– cells in LIP. Therefore, we compared the spike‐field coherence of the AE+ and AE– cells.
  For this analysis we performed multi‐taper spectral analysis, using Chronux toolbox (http://chronux.org; Bokil et al. 2010). The coherence is given by C(f) = S_12_(f)/√(S_11_(f)S_22_(f)), where S(f) is the spectrum with subscripts 1 and 2 referring to the simultaneously recorded spike train and LFP. We calculated coherence spectra in 300 msec sliding windows, stepped 50 msec, using 5 Slepian taper functions and a time bandwidth product equal to 3. The coherence was transformed to account for different numbers of trials between the groups (Bokil et al. 2007), that is, T(f) = tanh^−1^(C(f))‐1/(v_0_‐2), where v_0_ is the degrees of freedom; for our multi‐taper estimates, v_0_ = 2*K*N, where K is the number of tapers (5) and N is the number of trials. Comparison of AE+ and AE– groups was performed on the transformed coherence from all recording sites within the time‐frequency range where prominent coherence was previously observed during the delay period (Saalmann et al. 2007), namely the mean transformed coherence in a window of 400 msec before S2 onset in the 21–43 Hz frequency range (Wilcoxon Rank Sum test).
  We also investigated the association between spiking activity and the LFP in the time domain, by calculating the spike‐triggered average of the LFP (STA) during the late delay period (400 msec before S2 onset) in the beta to low gamma range of frequencies that were found to be related to top‐down modulation in our earlier study (Saalmann et al. 2007). We thus filtered the LFP in the 10–45 Hz band (zero phase‐shift 6‐pole Butterworth filter) and z‐scored it (i.e., subtracted its mean and divided the LFP by its standard deviation to normalize all LFPs to one scale). For each cell, we compared the peak‐to‐trough amplitude of the average LFP wave triggered from real spikes (i.e., LFP peak‐to‐trough surrounding the spike) to the distribution of the average LFP produced by taking the same number of random pseudospikes. The latter distribution was produced by calculating random STAs 1000 times and calculating peak‐to‐trough amplitudes in the same time window as for the real LFP wave. The STA amplitude from real spikes as well as from pseudospikes was calculated in a window ±55 msec around the spike, ignoring deflections of <12 msec duration. The real STA‐LFP wave was considered to be significantly modulated if its peak‐to‐trough amplitude was above 95% of the randomly taken STAs.

*Correct versus incorrect responses**.*** We compared neuronal activity of AE+ and AE– cells for correct and error trials, to address the question of whether the errors are related to poorer attention or poorer perceptual discrimination. An attentional deficit leading to errors may be reflected in a reduction in the attentional enhancement shown by the AE+ cells. On the other hand, the neuronal correlate of perceptual errors may be poorer orientation discrimination by the AE– cells on the particular trial. AE+ cells may thus show less attentional enhancement (i.e., reduced AEI) in the Match error trials, and AE– cells poorer orientation discrimination in Non‐Match error trials.
  As the number of error trials was always considerably smaller than the number of correct trials, we analyzed only population responses and did not perform these comparisons for individual cells. In some recording sessions, there were too few errors for these to be even included in our analysis. We thus used a limit of at least 4 error trials for each trial type before they could be used for the analysis. Thus, 16 cells from ‘Match Pr‐Pr’ trials were analyzed (mean number of error trials = 10, range: 4–17) and 19 cells from both types of Non‐Match trials, Pr‐Npr and Npr‐Pr (mean number of error trials = 21, range: 7–62).

## Results

We report here data from 47 LIP cells from two macaques that showed significant excitatory responses to neutral visual grating stimuli (i.e., response to S1 compared with the cell's spontaneous activity, Wilcoxon Signed Rank Test, *P *< 0.05). The majority of cells (29/47) in this sample recorded from LIP had been used in an earlier study, to study these cells' role in spatial attention and in top‐down modulation of responses in area MT (Saalmann et al. 2007). Here, we found that many (*n *= 24) of these 47 cells showed greater responses to the second grating in the task when the first grating had appeared at the same location, similar to the attentional enhancement shown in our earlier study (Saalmann et al. 2007). For the pooled sample of 47 cells, a significant, 29.22% attentional enhancement was measured in the “spatial and feature‐based attention” (i.e., ‘Match Pr‐Pr’) condition (*P *< 0.002) and a 22.70% enhancement in the “spatial attention” (i.e., ‘Non‐Match Npr‐Pr’) condition (*P *< 0.0001). As a control, our earlier study had shown that the attentional enhancement happened only when the monkeys had to attend to the visual stimuli, and not in a simple fixation task, in which the same visual stimuli were presented at the same locations, but the monkeys were cued to ignore them. In line with these neural responses, it can be inferred that the monkeys did orient their attention to the location of S1 during the rest of each trial, since any other strategy such as orienting to locations other than S1 could not have helped them to attain the low error rates (percentage correct was above 75% in the majority of blocks).

### Orientation selectivity and attentional enhancement

Significant attentional enhancement (Wilcoxon Signed Rank Test, *P *< 0.05) was observed in 24 individual cells out of the 47 LIP cells examined: the average attentional enhancement for this subset of cells was 51.06%. No significant differences were found between the AE+ and AE– groups in either spontaneous activity or magnitude of response to S1. To explore the relationship between attentional enhancement and orientation selectivity, we compared the OSI of the two groups of cells: those cells showing significant attentional enhancement (AE+) and those without (AE–). The data reported here are from 29 cells from Monkey 1, of which 12 were AE+ cells and 17 were AE– cells, and 18 cells from Monkey 2, of which 12 were AE+ cells and 6 were AE– cells.

**Figure 2:**
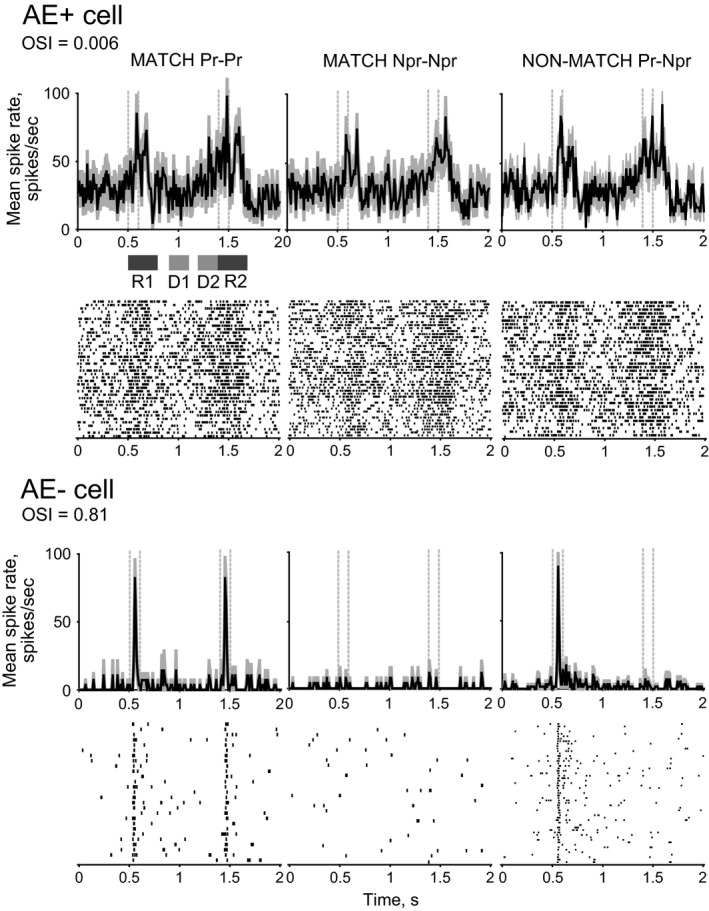
Peristimulus time histograms (PSTHs) and raster plots for 2 cells, top half showing responses of an AE+ cell, bottom half an AE– cell. Each column represents one trial type; first and second columns show ‘Match’ trials with 2 different orientations, either both preferred orientation (Pr‐Pr) or both nonpreferred (Npr‐Npr), respectively, and the third column represents ‘Non‐Match Pr‐Npr’ trials. Times of presentation of stimuli, S1 and S2, are indicated by dashed lines. Please also note that the orientation sensitivity index (OSI) calculated from responses to preferred and nonpreferred orientations presented as S1 are also indicated for each cell. The response regions (R1, D1, D2 and R2) used for all analysis are also indicated below the abscissa of the top left PSTH.

Figure 2 shows responses of a cell from each of the two cell groups under three different trial‐type conditions, to demonstrate the range of differences we observed among LIP cells. The top half shows peristimulus time histograms (PSTHs) and raster plots of an AE+ cell, and the bottom half PSTHs and raster plots of an AE– cell. The first two columns show responses to the two orthogonal orientations in Match trials, demonstrating poor orientation sensitivity, but significant attentional enhancement for Cell 1, and vice versa for Cell 2. The last column shows Non‐Match trials with preferred S1 and nonpreferred S2 grating stimuli (‘Non‐Match Pr‐Npr’). Cell 1 shows dominance of attentional enhancement without noticeable orientation preference and cell 2 shows dominance of orientation preference without any attentional enhancement.

**Figure 3:**
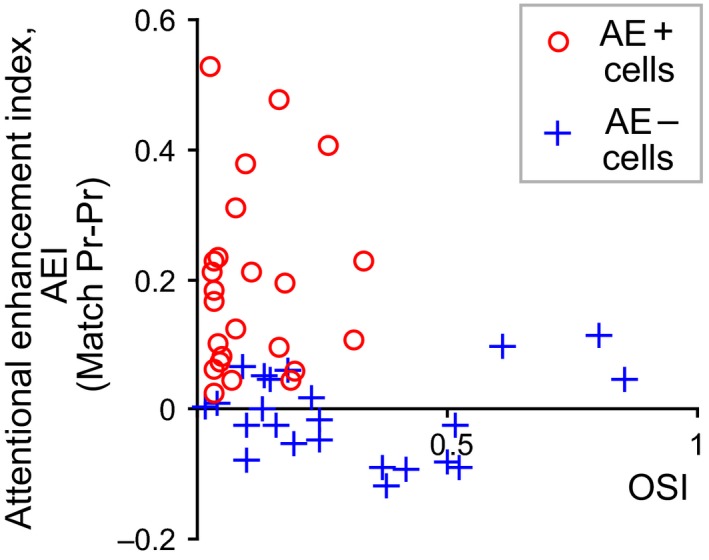
Relationship between orientation selectivity (OSI) and attentional enhancement (AEI). AEI is plotted against OSI for ‘Match Pr‐Pr’ trials. Crosses represent AE– cells and circles AE+ cells. See text for how OSI is measured.

**Figure 4:**
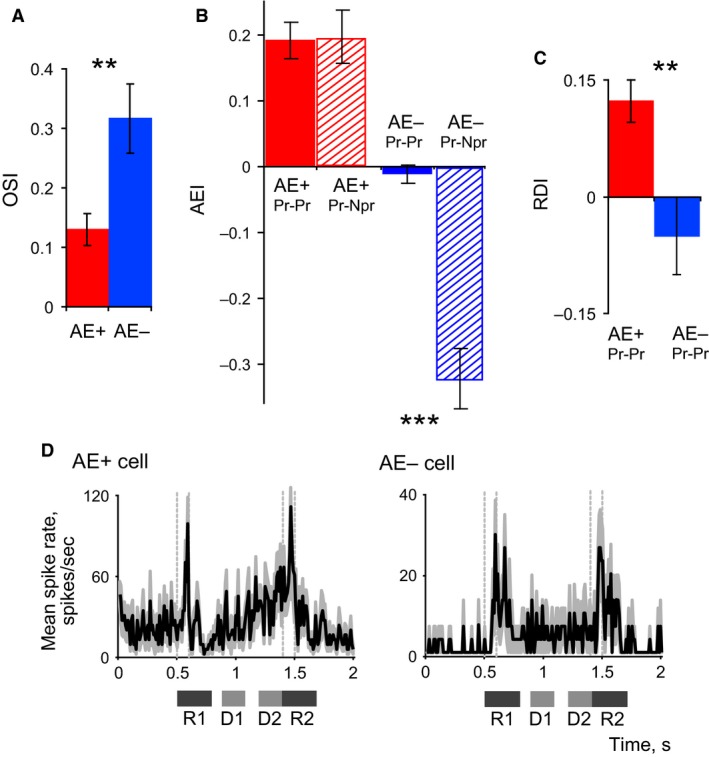
(A) Mean (±SEM) of orientation selectivity index (OSI) in the two groups of LIP cells, the left bar representing the AE+ group and right bar the AE– group. (B) Mean attentional enhancement indices shown for AE+ and AE–cells for ‘Match Pr‐ trials and ‘Non‐Match Pr‐Npr’ trials. (C) Mean (±SEM) of ramping up index during the delay period (RDI) seen in the AE+ and AE– LIP cells. In A to C, **P < 0.01 and ***P < 0.001. (D) Peristimulus time histograms (PSTHs) of an AE+ and an AE‐ cell, demonstrating the differences between the cells in the response during the delay period: the AE+ cell shows a ramping up of activity, whereas the AE‐ cell shows stable, moderately elevated activity. R1 and R2 are the regions for which responses to S1 and S2 were calculated and D1 and D2 represent the two delay periods chosen for analysis.

The two cells shown in Figure 2 demonstrate, for the sake of clarity, only two of the more extreme examples, but the whole sample is represented in the scatter plot of Figure 3. The figure shows data from ‘Match Pr‐Pr’ trials separately for the two groups, one which shows significant attentional enhancement (AE+ cells, open circles) and the other showing no such enhancement (AE– cells, crosses). Please note that the OSI data shown in the abscissa are calculated from evoked responses to S1 (i.e., a grating of preferred orientation presented as the first stimulus) and thus relate to the orientation tuning unaffected by attention or any working memory components. All AE+ cells (open circles) show generally poor orientation selectivity, but the AE– cells (crosses) span the whole spectrum with many showing considerable orientation sensitivity. The mean orientation selectivity of AE+ cells was significantly lower than that of AE– cells (Fig. 4A; Wilcoxon Rank Sum Test, *P *< 0.01).

The AEI of AE+ cells in ‘Match Pr‐Pr’ trials was also similar to that in ‘Non‐Match Pr‐Npr’ trials (Fig. 4B, left; Wilcoxon Signed Rank Test, *P* > 0.5). In fact, no significant difference was observed between AEIs of ‘Match Pr‐Pr’ and either Non‐Match condition (Npr‐Pr or Pr‐Npr), indicating that AE+ cells exhibit the same degree of response enhancement to the second stimulus irrespective of the orientation of the first stimulus presented to the RF. However, this relationship was very different in the case of AE– cells (Fig. 4B, right). They showed no attentional enhancement, but had a significantly lower AEI in Non‐Match ‘Pr‐Npr’ trials compared to ‘Match Pr‐Pr’ trials (Fig. 4B; Wilcoxon Signed Rank Test, *P *< 0.001). This marked change in their response to the second stimulus is thus attributable entirely to the feature selectivity of these AE– cells.

We also tested whether there was an effect of featural memory or feature‐based attention on the evoked‐responses of AE+ and AE– cells to the second stimulus (S2). We did this by measuring: (1) any difference in response to the preferred S2 in the RF (for 300 msec from S2 onset) when the first stimulus, S1, in the RF was of the preferred orientation versus the nonpreferred orientation; and (2) any difference in response to the preferred S2 in the RF when S1 outside the RF was of the preferred orientation versus the nonpreferred orientation. Especially in case b, featural memory or feature‐based attention would be evident as a difference in S2 response resulting from the featural information contained in S1. We performed the analysis for all cells that had trials in all of the aforesaid conditions by normalizing the S2 response (R2, as defined in Methods and Materials) to the neutral stimulus (preferred S1 in RF). For trials with both S1 and S2 on the RF (case a), the featural information in S1 produced no significant difference in S2 responses for either AE+ cells (Wilcoxon Signed Rank test, *n *= 24, *P *= 0.09) or AE– cells (Wilcoxon Signed Rank test, *n *= 23, *P *= 0.2). Similarly, for trials with S1 outside the RF and S2 inside the RF (case b), the orientation of S1 had no significant influence on the S2 response from LIP cells (AE+ cells: Wilcoxon Signed Rank test, *n *= 24, *P *= 0.62; AE– cells: Wilcoxon Signed Rank test, *n *= 13, *P *= 0.13). Thus, featural memory or attention had little influence on the evoked‐response to S2 in our experimental paradigm. It is, however, possible that other paradigms may be able to reveal direct modulation of sensory responses based upon featural history.

### Delay period activity in AE+ and AE– cells

Many LIP cells show a gradual increase in maintained activity (ramping) in expectation of a visual event (Colby et al. 1996; Eskandar and Assad 2002; Janssen and Shadlen 2005), including during the delay period in our DMS task (Saalmann et al. 2007), especially just prior to the presentation of the second stimulus. We compared the average spike rates between two time windows, one toward the start, and the other toward the end, of the delay period (D1 and D2 in Fig. 1B). The ramping index, RDI (see Methods and Fig. 4C) showed that such ramping during the delay period was significantly higher for the AE+ cells (Wilcoxon Rank Sum Test, *P *= 0.004). The mean increase of D2 compared to D1 in AE+ group was 34.8% (*P *< 0.001), whereas no significant difference between D1 and D2 was found for the AE– group (*P *= 0.37).

While the gradual increase in activity during the delay period for AE+ cells may be related to focal spatial attention, a more sustained activity during the whole delay period, or at least during the early part of the delay on the heels of the response to S1, may suggest a role in working memory, as indeed has been shown in human imaging studies (Ikkai and Curtis 2011). Therefore we compared activity during the whole delay period to background activity (B) prior to S1. Both AE+ and AE‐ cells demonstrated elevated delay period responses (Wilcoxon signed rank test, *P *< 0.05). However, when we compared early delay period (D1) and late delay period (D2) separately to background (B), the results were very different for the two cell types (Fig. 4D). AE+ cells had significant elevation of activity only for the late delay period (*P *= 0.001), while AE‐ cells had significantly elevated activity in both D1 and D2 (*P *= 0.006, *P *< 0.001). However, these results do not necessarily indicate that AE‐ cells have stronger delay period elevation overall: while AE‐ cells had a mean elevation of 52% for D1 and 51% for D2 periods, the AE+ group had only a 30% increase for D1 but a 81% increase for D2 periods. This finding, together with the results on ramping, shows that AE– cells maintain a relatively constant but moderately elevated response for the whole delay period, consistent with a role in feature memory and discrimination; whereas AE+ cells have a strong rise in activity as time approaches S2, consistent with a role in spatial attention.

### Local synchrony as seen in spike‐field coherence and spike‐triggered LFP average

**Figure 5:**
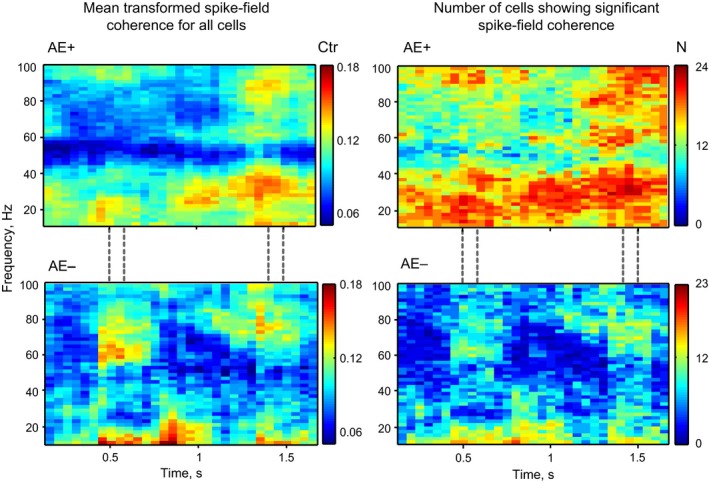
Comparison of spike‐field coherence for AE+ (top row) and AE– (bottom row) cell groups. Coherence calculated in 300 msec sliding windows, in steps of 50 msec across the trial (for example, data plotted at 0.5 sec reflects coherence in the window between 0.35 sec and 0.65 sec). Left part of the figure shows mean transformed coherence (Ctr) for AE+ and AE‐ groups. Mean transformed coherence is color‐coded. Right part demonstrates the number of cells (*N*) that show significant coherence in each time‐frequency window in the frequency range 10–100 Hz in steps of 2 Hz for the group of 24 AE+ and 23 AE– cells. Number of cells showing significant coherence color‐coded.

Figure 5 demonstrates the transformed spike‐field coherence for the AE+ (top row) and AE– (bottom row) cell groups. The left column demonstrates the population mean transformed coherence (Ctr) for the AE+ and AE– groups, and the right column shows the number of cells having a coherence value significantly above 0 for each time‐frequency point (jackknife and theoretical error calculations produced similar results; Jarvis and Mitra 2001). There are 24 AE+ cells, so the right top panel has a range from 0 to 24 cells (N); and the right bottom panel shows the group of 23 AE– cells. Coherence level for the AE+ group in the high beta to low gamma range (21–43 Hz) is significantly higher (Wilcoxon Rank Sum Test, *P *< 0.05) in the late delay period (400 msec interval before S2 onset) compared with the AE– group. This suggests that AE+ cells are firing in synchrony with their neighbors in precisely the high beta to low gamma frequency range that has been shown to be associated with attentional feedback, in the FEF‐LIP projection (Buschman and Miller 2007) as well as in the LIP‐MT projection (Saalmann et al. 2007). Spiking activity of AE– cells did not show a similar synchronization with the LFP. Instead, there was a small increase in high gamma activity (>50 Hz) seen around S1 and S2 with AE– cells, which may reflect stimulus‐driven responses, consistent with suggestions that such gamma activity may reflect bottom‐up processes (e.g., 40–90 Hz, in Van Kerkoele et al. 2014).

**Figure 6:**
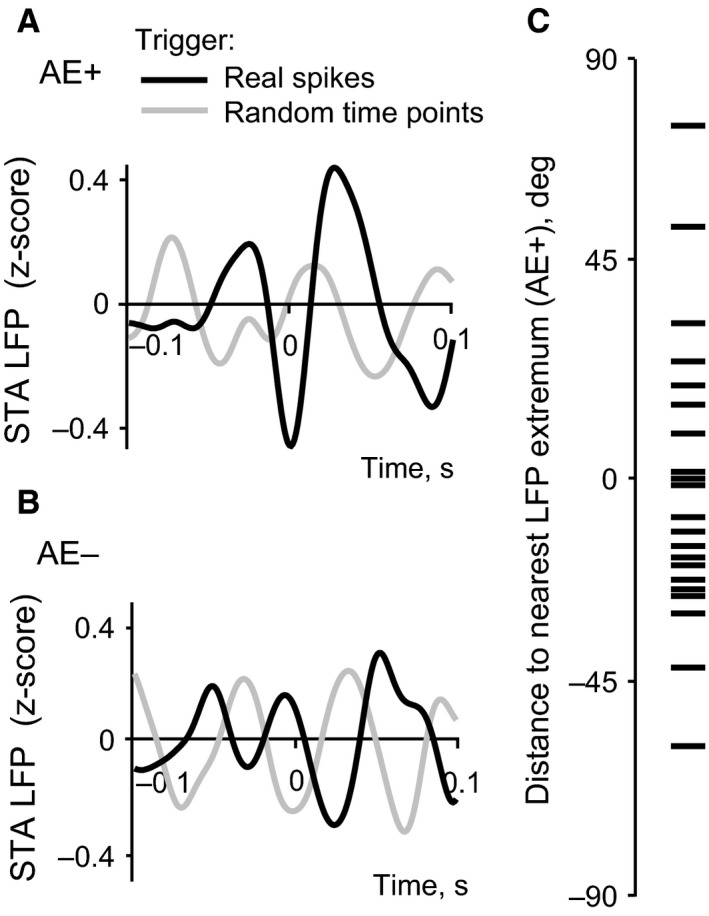
Spike‐triggered LFP average (STA). (A) STA example for an AE+ cell with significant LFP modulation around spikes (black line) and the LFP triggered by random pseudospikes (gray line). Spikes occur at the troughs of the LFP‐oscillation. The average based on random time points has lower amplitude of modulation. (B) STA example for AE‐ cell without significant LFP modulation around spikes (black line) and the LFP triggered by random time points (gray line). Both averages have similar amplitudes of modulation around the trigger (0). (C) Distribution of distance in time of the spikes to the nearest LFP extremum (peak/trough) for AE+ cells. The distance is given as a percentage of the peak‐to‐peak or trough‐to‐trough interval surrounding the above extremum. +% denotes spikes occurring after the extremum and ‐% denotes spikes occurring before the extremum.

Figure 6 shows the spike‐triggered LFP average (STA LFP) in the case of a typical AE+ cell (A) and a typical AE– cell (B). We found that 21 of 24 AE+ cells had a significant STA LFP response, compared to only 7 out of 23 cells in the AE– group. Thus, as a population, the LFPs recorded at the site of AE+ cells had significant modulation associated with spikes, whereas the LFPs from the site of AE– cells did not (Fisher Exact Test *P *< 0.001). AE+ cells tend to fire in the vicinity of the, mostly low, extrema of the LFP‐wave, with 15 out of 21 cells firing close to the trough and only 3 close to the peak. An example of significant STA LFP for an AE+ cell which fired at the minimum of the LFP curve is presented in Figure 6A. For all cells with significant STA, we also determined the distance in time between the spikes and the nearest extremum of the LFP wave in relation to the peak‐to‐peak or trough‐to‐trough distance of the wave surrounding the spike. The distribution of the distances to the nearest extremum for all AE+ cells is shown in Figure 6C. The coupling of the majority of attention‐selective LIP cell responses to the LFP cycle indicates their synchronization, which would help provide synchronized attentional feedback to other visual areas.

### Attention and perception in error trials

**Figure 7:**
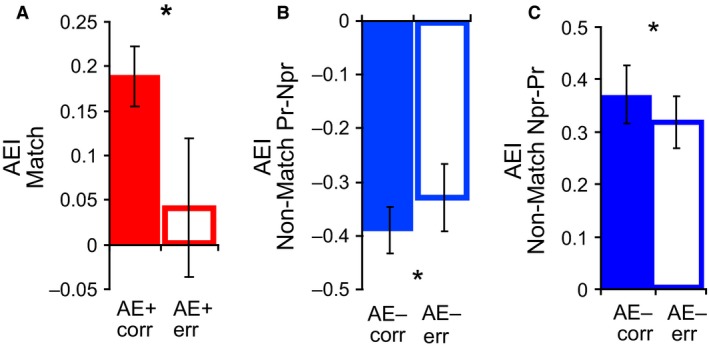
(A) Mean (±SEM) of attentional enhancement in AE+ group for correct and error “Match Pr‐Pr” trials. Filled bars represent correct trials, empty bars error trials. (B) Mean (±SEM) of attentional enhancement in AE– group for correct and error “Non‐Match Pr‐Npr” trials. (C) Mean (±SEM) of attentional enhancement in AE– group for correct and error “Non‐Match Npr‐Pr” trials. *P < 0.05.

Response errors in the delayed match‐to‐sample task could be due to either reduced attention or poor orientation discrimination. In the AE+ cell group, the AEI of ‘Match Pr‐Pr’ trials was significantly higher for correct trials compared to error trials (Wilcoxon Signed Rank Test, *N *= 16, *P *< 0.05; Fig. 7A), suggesting that the weaker attentional enhancement of AE+ cells in some trials could be related to the errors made by the animal. No such difference was observed in the case of AE– cells for ‘Match Pr‐Pr’ trials (*N *= 15, *P *= 0.21).

To probe possible changes in orientation discriminability of AE‐ cells in error trials, we again calculated AEI for Non‐Match trials. Note that, as shown earlier, the AEI for ‘Match Pr‐Pr’ trials reflects any attentional modulation (for which there was little for AE‐ cells); whereas calculating the AEI measure for AE– cells in Non‐Match trials largely reflects orientation selectivity (as S1 and S2 have different orientations in this scenario). The AE‐ cell group showed a significant difference in this measure between correct and erroneous Non‐Match trials with different S1 and S2 orientations. Discrimination of different orientations by AE– cells was moderately, yet significantly, poorer for error trials in both ‘Non‐Match Pr‐Npr’ (*N *= 19, *P *= 0.04; Fig. 7B) and ‘Non‐Match Npr‐Pr’ (*N *= 19, *P *= 0.03; Fig. 7C) conditions. This indicates that reduced orientation discrimination by AE– cells could also potentially contribute to behavioral errors. In comparison, AE+ cells did not show any change in orientation discriminability for erroneous Non‐Match trials when S1 had the preferred orientation (Pr‐Npr, *N *= 15, *P *= 0.78) nor when S2 had the preferred orientation (Npr‐Pr, *N *= 16, *P *= 0.87).

## Discussion

### Neuronal signatures of spatial attention and feature selectivity in different sets of LIP neurons

A possible critical role for LIP in controlling attention in macaques has been suggested by single neuron studies (e.g., Colby et al., 1996; Colby and Goldberg 1999; Bisley and Goldberg 2003, 2010; Greenberg et al. 2010; Bisley et al. 2011; Saalmann et al. 2007). Such an attentional role is further supported by macaque imaging experiments, which have shown a correlation between LIP activity and the number of distractors in a visual search task that increases the demands on attentional resources (Atabaki et al. 2014). However, evidence has been emerging that there is some degree of heterogeneity in the neuronal population of LIP (Ogawa and Komatsu 2009; Premereur et al. 2011). Consistent with this, our present results have identified a relative segregation in the function of LIP neurons with regard to two fundamental properties: LIP cells tend to exhibit either significant feature (namely, orientation) selectivity in their responses (AE– cells) or significant attentional modulation (AE+ cells), but not both. The finding of feature‐sensitive LIP neurons is consistent with earlier reports of feature selectivity in LIP (Sereno and Maunsell 1998; Saalmann et al. 2007; Ogawa and Komatsu 2009; Mendoza‐Halliday et al. 2014; Subramanian and Colby 2014), but our present results additionally suggest that this featural information may be used by a different group of LIP neurons (AE+ cells) to direct spatial attention. As per the “Guided Search” model of attention, a saliency map needs to be constructed first in order to direct serial spatial attention. Thus, feature‐selective cells would act at the first stage and attention‐related cells at a later stage of information processing. It is noteworthy that the cells which show the greatest response closer to the second stimulus during the delay period (i.e., high ramping indices) are in fact the AE+ cells. Such pre‐stimulus activity is likely to facilitate the cell's response to the second stimulus in the delayed match‐to‐sample task, leading to the top‐down facilitation of responses in the topographically corresponding region of earlier visual areas such as MT (Saalmann et al. 2007; Herrington and Assad 2010).

Our results are consistent with an earlier study using a different experimental paradigm – a visual search task with the target defined in either the shape or color dimension – that reported three cell types in LIP (Ogawa and Komatsu 2009). One cell type showed largely a spatial response irrespective of the specific features or relevant featural dimension of the target; a second cell type responded preferentially to a specific target feature (e.g., rectangle, circle, black or white) regardless of whether the relevant target dimension was shape or color; and a third cell type responded preferentially to a specific feature when it belonged to the relevant target dimension of color or shape (Ogawa and Komatsu 2009). In addition to confirming their basic observation of cell categories in LIP (one spatial category, and the others related to featural information), our delayed match‐to‐sample paradigm enabled us to quantify featural information in working memory during the delay period, which was also unaffected by any saccadic preparation.

The poor local synchrony of AE– cells with their neighboring cells, gauged from the spike‐field coherence and STA LFP during the late delay period, suggests little clustering of LIP (AE–) cells that are selective for similar visual features. This may well suit the reconfigurations of LIP's network activity necessary for higher cognitive functions, such as categorizing stimuli according to behavioral demands (Freedman and Assad 2006). In contrast, the development of local synchrony for the AE+ cells during the delay period is consistent with neighboring cells representing a particular location in visual space, reflecting the rough topographic organization of LIP (Blatt et al. 1990; Ben Hamed et al. 2001). The spike phase‐locking of most AE+ cells to the trough of the LFP is consistent with a human study (Jacobs et al. 2007) of spike–LFP relationships in parietal cortex (and other cortical areas) during a visual spatial task. This synchronization of AE+ cells may thus enhance feedback about behaviorally relevant locations from LIP to other areas of the visual cortex (Saalmann et al. 2007).

### Possible transition from working memory to attention during the delay period in LIP

Separate analysis of neuronal activity of AE+ and AE– cells during the delay period revealed another fundamental difference between these two groups of cells: AE+ cells showed ramping up of delay period activity, whereas AE‐ cells showed a relatively constant and moderate elevation of delay period activity. First, let us consider the response of AE+ cells and the possible contribution of not only attention, but also simple repetition of a visual stimulus, working memory, or preparation for a specific motor response. We believe that the response enhancement of AE+ cells is largely due to attention for a number of reasons. (1) We found that AE+ cell responses to the second stimulus in our paradigm were modulated only by the spatial location of the first stimulus, not by the orientation of the first stimulus. This was true also for the AE– cells. (2) This and an earlier study (Saalmann et al. 2007) using the same monkeys found significant ramping in LIP cells only toward the late delay period just prior to the upcoming second stimulus. Because the activity of the AE+ cell group was not significantly elevated early in the delay period, their spike rate could not support working memory during this time. (3) The late delay period also exhibited an oscillation in the beta to low gamma range that was synchronized with activity in area MT (Saalmann et al. 2007), helping LIP feedback to facilitate the attentional enhancement of MT neurons. The poor feature selectivity shown in this feedback from LIP better fits a spatial attention interpretation rather than featural memory. (4) The LIP activity for the second stimulus here followed the same time course as the classical attentional blink, as shown in another study, using the same two monkeys (Maloney et al. 2013). (5) The two monkeys had also been trained on a passive fixation task, in which the same series of visual stimuli were presented as in the DMS task, but the monkeys were cued to ignore them and perform a simple fixation. The same stimuli in this fixation task did not produce the enhanced response to the second stimulus (see Supplementary Figure S1 in Saalmann et al. 2007). The response enhancement of the AE+ cells only occurred when the monkey had to pay attention to the stimuli, suggesting that it was more likely due to attention than any bottom‐up repetition or priming effect.

Delay activity of AE‐ cells, on the other hand, revealed a likely involvement in working memory. Though moderate, there was a significant increase in the maintained discharge for the group of AE‐ cells during the delay period (both early and late) compared to the background discharge prior to the first stimulus. Such an increased responsiveness for AE‐ cells occurred in the early delay period (D1) immediately after the response to the first stimulus, unlike the AE+ cells, which showed such an increase only late in the delay period (D2). However, AE‐ cells, unlike the AE+ cells, did not show the gradual increase in their discharge (ramping) in the period (D2) immediately prior to the second stimulus. These results suggest that AE‐ cells may perform a working memory function immediately following S1 and possibly throughout the delay period. Later in the delay period, this memory signal could be transmitted to AE+ cells at the time when the LIP priority map needs to be primed for the selective enhancement of incoming sensory inputs. Recent fMRI work reveals persistent activity around the human intraparietal sulcus, a region that shares a number of response characteristics with macaque LIP, related to both maintenance of working memory representations and spatial attention (Ikkai and Curtis 2011). Our results are not only consistent with this, but elucidate its cellular basis, showing that the two functions are represented in two groups of cells with a timing that is highly suggestive of the working memory mediated by one group (AE‐ cells) leading to the spatial attention mediated by the other (AE+ cells). However, this does not preclude the involvement of other areas such as prefrontal or visual cortex in maintaining working memory representations essential for tasks such as the DMS.

### Role of LIP in top‐down selective attention

The idea that the parietal cortex may use spatial and/or featural signals to determine a purely spatial priority for its feedback to earlier visual areas (Vidyasagar 1999; Bullier 2001; Deco and Rolls 2004) is consistent with models postulating a spotlight of attention that selects object locations for processing in a serial fashion (Treisman and Gelade 1980; Treisman 1988). Treisman's early models saw a further development in the Guided Search model (Wolfe 1994, 2007) which articulated a two‐stage process, with a feature‐based parallel stage preceding the serial, spatial allocation of attention, in contrast to the one‐stage models (McElree and Carrasco 1999; Baldassi and Burr 2000; Baldassi and Verghese 2002). Such feedback, highlighting locations of interest, is supported by the finding that attentional enhancement of MT responses due to putative LIP drive was spatial, without a significant featural component (Saalmann et al. 2007). In this study, we report the neuronal basis for a possible two‐stage process in LIP that could potentially mediate top‐down attentional modulation. The two groups of LIP cells found in this study are together capable of extracting spatial priorities on the basis of featural cues. This allows spatial attentional feedback, possibly extending to the primary visual cortex (Vidyasagar 1998; McAdams and Reid 2005; Vidyasagar and Pigarev 2007), in accordance with schemes (Vidyasagar 1999; Bullier 2001) that provide the neural framework for the Guided Search model (Wolfe 1994, 2007). These schemes postulate a dorsal stream spatial feedback to the primary visual cortex, which gates the information that enters the ventral stream for detailed object identification. A dorsal stream advantage for spatial localization of objects, as well as the faster access to visual information through its magnocellular input, can together lead to a sufficiently rapid feedback to the primary visual cortex, which selectively facilitates visual responses at a fixed location represented in V1 and gates the more slowly arriving parvocellular‐mediated visual signals to be channeled to the ventral stream (Vidyasagar 1999; Vidyasagar 2013; Bullier 2001; Laycock et al., 2007). However, LIP also has direct connections with ventral stream areas (Blatt et al. 1990), which allow for signal gating at levels beyond V1.

**Figure 8:**
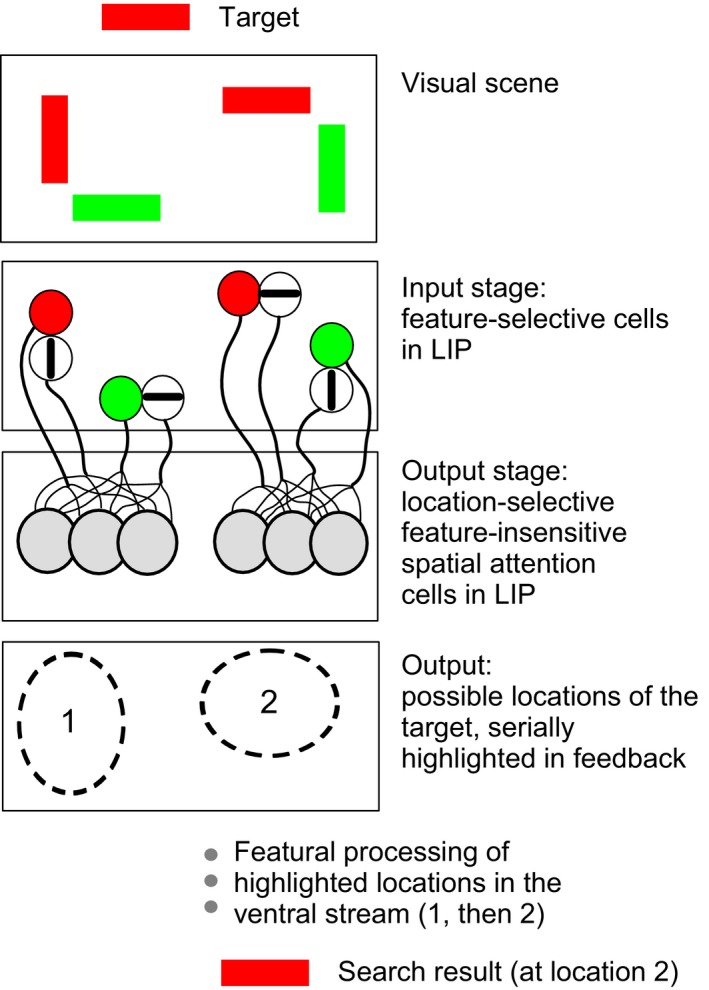
Diagrammatic representation of the two‐stage model of LIP's role in spatial attention. Top panel shows a sample of items in the visual scene containing a target (red horizontal bar) among distractors. Second panel represents the first (input or intermediate) stage of feature‐selective cells activated by one feature (say, red items). In the third panel, the prioritized ‘modules’ of the first stage that each cause, in a serial fashion, synchronized oscillations within a cluster of cells that represent a spatial location. The last panel represents the locations that are serially highlighted by the feedback from LIP to earlier visual areas. Finally the object at each highlighted location is serially processed in the ventral stream. See text for further details of the scheme.

Figure 8 describes a possible neuronal scheme that can be applied not just to our DMS task, but also more generally to selective attention, such as in common visual search situations. It proposes that the two types of cells (AE+ and AE–) in LIP occupy different stages in the parietal circuitry and thus may be located in different cortical laminae, performing fundamentally different functions. The feature‐selective cells are first activated – for example, with this first stage selecting all locations that have a red item – either in parallel, by the appropriate stimuli in the visual scene, or serially, under the influence of the prefrontal cortex. The sustained activity of these feature‐selective cells, transmitted serially to the AE+ cells representing the same spatial location, would activate the local assembly of mutually interconnected AE+ cells. The combined activation of these AE+ cells would lead to their attentional enhancement, the synchronized neuronal activity evident in the spike‐field coherence, and to the increased response to the visual stimulus seen in topographically corresponding parts of earlier visual areas. Such localized activation in early visual areas facilitated by the feedback is likely to be the correlate of the ‘spotlight of attention’ (Treisman 1988; Vidyasagar 1998; Brefzynski and DeYoe 1999; McAdams and Reid 2005; Saalmann et al. 2007), which may underlie the integrated processing of attributes that belong to the attended object (Vidyasagar 1999; Bullier 2001; Deco and Rolls 2004). The experimental paradigm used in this study, though not a visual search paradigm, has nevertheless the essential ingredients that potentially implicate the two categories of cells (AE+ and AE‐) in directing attentional feedback. For the sake of clarity, the model represented in Figure 8 does not specifically show a route to the output AE+ cells for allocating attentional priorities in the absence of a specific visual stimulus, but can be expanded to include an input based upon a past cue, memory or other executive signal from areas such as the prefrontal cortex (Ibos et al. 2013).

One open question is whether the tuning of the feature‐selective AE– cells is influenced by task demands. In the light of a recent experiment by Ibos and Freedman (2014), which showed flexible integration of color and direction signals in LIP neurons, it is possible that the sample orientation in our task may lead to sharper tuning of the AE– cells on a short time scale to enable a more efficient response on each trial.

The difference in the roles of AE+ and AE‐ cells in LIP function is further underscored by their respective responses in error trials. The response of AE+ cells in error trials, on average, showed poorer attentional enhancement, which would likely perturb attentional feedback to visual cortex. Conversely, AE– cells showed poorer orientation discrimination in error trials. In the neuronal scheme outlined above, the responses of AE‐ cells in error trials could also lead to perturbed feedback to the visual cortex, via the influence of AE‐ cells on the AE+ cells.

A largely spatial, rather than featural, spotlight from LIP on early visual areas is functionally meaningful, since detailed analysis of a selected object of interest would require processing of many of the object's features. Note that any object has a unique location in space, whereas a feature can be shared by different objects. Therefore, in a cluttered scene, it would be computationally less taxing to lead with a spatial search than to search feature‐by‐feature until all relevant features of an object are found. Furthermore, object transformations, such as rotation, may lead to changes in the features that need to be processed. However, changes in object location may be missed by a serial search dominated largely by spatially localized feedback signals, and there is human psychophysical evidence suggesting that this is indeed the case (Horowitz and Wolfe 1998). Furthermore, in a motion task using both spatial and featural cues, human subjects' performance was determined more strongly by spatial attention rather than by feature‐based attention (Verghese et al. 2013). The concept of a strongly spatial nature of LIP output is also supported by a study that showed local inactivation of LIP leading to predominantly spatial deficits despite the presence of nonspatial modulatory signals in LIP neurons (Balan and Gottlieb 2009). Thus, it would be parsimonious to have one final common pathway of LIP output corresponding to a particular spatial location, which can potentially be triggered by different stimuli and contingencies.

In many real life situations, attention needs to be maintained at a specific location, even if the object is no longer present or the object's features are changing. Further, in typical visual search situations, there may be a subset of possible targets to be serially inspected based upon a common feature they share. We suggest that these different demands may be well serviced by a LIP priority map, which is driven at the input level by both bottom‐up and executive signals to LIP (i.e., from frontal cortex) that are at least moderately feature‐sensitive. At the output level, primarily spatial LIP responses would modulate representations of specific locations or objects in earlier sensory areas.

## Conflict of Interests

Authors state no competing interests.
